# Inhibition of DNA Methylation With Zebularine Alters Lipopolysaccharide-Induced Sickness Behavior and Neuroinflammation in Mice

**DOI:** 10.3389/fnins.2018.00636

**Published:** 2018-09-18

**Authors:** Stephanie M. Matt, Jalisa D. Zimmerman, Marcus A. Lawson, Angela C. Bustamante, Monica Uddin, Rodney W. Johnson

**Affiliations:** ^1^Neuroscience Program, University of Illinois at Urbana-Champaign, Urbana, IL, United States; ^2^Animal Sciences Laboratory, Department of Animal Sciences, University of Illinois at Urbana-Champaign, Urbana, IL, United States; ^3^Carl R. Woese Institute for Genomic Biology, University of Illinois at Urbana-Champaign, Urbana, IL, United States; ^4^Department of Psychology, University of Illinois at Urbana-Champaign, Urbana, IL, United States; ^5^Integrative Immunology and Behavior Program, University of Illinois at Urbana-Champaign, Urbana, IL, United States

**Keywords:** microglia, neuroimmunology, *Il-1β*, DNMT inhibitor, DNA methylation, mouse models

## Abstract

Activity of DNA methyltransferases (DNMTs), the enzymes that catalyze DNA methylation, is dynamically regulated in the brain. DNMT inhibitors alter DNA methylation globally in the brain and at individual neural plasticity-associated genes, but how DNMT inhibitors centrally influence lipopolysaccharide (LPS)-induced neuroinflammation is not known. We investigated whether the DMNT inhibitor, zebularine, would alter sickness behavior, DNA methylation of the *Il-1β* promoter and expression of inflammatory genes in hippocampus and microglia. Contrary to our hypothesis that zebularine may exaggerate LPS-induced sickness response and neuroinflammation, adult mice treated with an intracerebroventricular (ICV) injection of zebularine prior to LPS had surprisingly faster recovery of burrowing behavior compared to mice treated with LPS. Further, genes of inflammatory markers, epigenetic regulators, and the microglial sensory apparatus (i.e., the sensome) were differentially expressed by zebularine alone or in combination with LPS. Bisulfite pyrosequencing revealed that ICV zebularine led to decreased DNA methylation of two CpG sites near the *Il-1β* proximal promoter alone or in combination with LPS. Zebularine treated mice still exhibited decreased DNA methylation 48 h after treatment when LPS-induced sickness behavior as well as hippocampal and microglial gene expression were similar to control mice. Taken together, these data suggest that decreased DNA methylation, specifically of the *Il-1β* promoter region, with a DNMT inhibitor in the brain disrupts molecular mechanisms of neuroinflammation.

## Introduction

Microglias are long-lived resident immune cells of the brain that show limited turnover (Ransohoff and Perry, 2009). They develop early in embryogenesis in the embryonic yolk sac and migrate to the central nervous system (CNS) where they remain and are rarely replaced (Ginhoux et al., 2010). Microglias are far from inactive and many recent *in vivo* observations have shown that microglia extend their processes to actively scan the microenvironment (Nimmerjahn et al., 2005; Wake et al., 2009). Despite the dynamic role of microglia in maintaining homeostasis, their long-lived nature and general inability to be replaced by circulating peripheral cells makes them particularly sensitive to oxidative stress, DNA damage, and a lifetime of inflammatory insults. Peripheral macrophage subtypes express different patterns of genes after stimulation with lipopolysaccharide (LPS) that is linked to environmental influence of distinct epigenetic modifications during their differentiation (Kittan et al., 2013). However, little is known about the epigenetic pathways involved in the modulation of inflammatory genes in the brain and microglia. As the immune system needs to respond to rapidly changing environmental cues, the molecular regulation of inflammatory responses in the brain is also a likely target for epigenetic regulation (Garden, 2013).

DNA methylation of pro-inflammatory cytokines such as *Il-1β* is a mechanism that regulates microglial reactivity and could be a therapeutic target for regulating microglia throughout the lifespan. One particularly important study determined that sirtuin 1 deficiency in aging microglia is associated with increased *Il-1β* transcription and decreased methylation of CpG sites within the *Il-1β* proximal promoter (Cho et al., 2015). More recently, findings from our lab (Matt et al., 2016) indicated that aged mice had decreased methylation of the *Il-1β* gene promoter in primary microglia basally or following systemic LPS that is associated with increased *Il-1β* mRNA. Further, the DNMT inhibitor 5-azacytidine increased *Il-1β* gene expression and decreased DNA methylation of primary microglial cells.

DNA methylation and demethylation are dynamically regulated in the brain (Kundakovic et al., 2009; Roth et al., 2009), and it has been demonstrated that DNA methylation changes can happen in as quickly as 1 h (Miller and Sweatt, 2007). The reversible nature of epigenetic aberrations contributing to human diseases makes them desirable therapeutic targets. 5-Aza-2′-deoxycytidine and 5-azacytidine are DNMT inhibitors that are potential chemotherapeutic agents for cancer, and have been approved for treating myelodysplastic syndrome (Copeland et al., 2010). Both drugs act by incorporating into DNA where they bind and sequester DNMTs, which causes prevention of the maintenance methylation (Gnyszka et al., 2013). However, both compounds are chemically unstable and toxic. Zebularine is a stable nucleoside analog of cytidine that is a less toxic DNMT inhibitor and the first drug in its class that can reactivate an epigenetically silenced gene by oral administration (Cheng et al., 2003). Moreover, zebularine is comparable to 5-aza-2′-deoxycytidine and 5-azacytidine in terms of its pattern of DNA demethylation (Balch et al., 2005; Griffin et al., 2016).

A significant amount of research has utilized intracerebroventricular (ICV) zebularine injections in rodent models, such as a cocaine-induced behavioral sensitization model (Anier et al., 2010), and an ischemic brain injury model (Dock et al., 2015), to determine the relationship between DNA methylation status and disease. Since DNMT inhibition was able to demethylate the *Il-1β* gene promoter and subsequently increase *Il-1β* gene expression *in vitro* (Matt et al., 2016), the objectives of this study were to investigate whether central DNMT inhibition by zebularine causes exaggerated neuroinflammation in microglia and hippocampus. We hypothesized that central DNMT inhibition would lead to decreased *Il-1β* DNA methylation and heightened pro-inflammatory gene expression in adult mice as well as prolonged sickness behavior following central immune stimulation with LPS. Additionally, with the recent discovery of the microglial sensome (Hickman et al., 2013), a unique group of transcripts encoding proteins for sensing endogenous ligands and microbes, we hypothesized zebularine would alter genetic expression of sensome genes in microglia. Last, since DNA methylation affects other epigenetic processes such as histone modifications (Fuks, 2005), we predicted zebularine would change expression of epigenetic regulator genes within microglia.

## Materials and Methods

### Animals

Adult (3 to 6-month-old) male *C57BL*/*6* mice (Jackson Laboratory, Bar Harbor, ME, United States) were individually housed in a temperature-controlled environment with a 12-h reversed-phase light/dark cycle (lights on 21:00 h). Mice were allowed to acclimate to these conditions for at least 3 weeks before being stereotaxically implanted with a guide cannula (Plastics One, Roanoke, VA, United States) placed to extend 1 mm dorsal to the lateral ventricle, as previously described (Lawson et al., 2013). In brief, mice were deeply anesthetized with an intraperitoneal (IP) injection of ketamine, xylazine, and buprenorphine (100, 10, and 0.05 mg/kg, respectively) all at 100 μl/10 g body weight and the surgical site was shaved and sterilized. Cannulae were placed at 1.5 mm lateral, 0.6 mm posterior, and 1.3 mm dorsal with respect to bregma. Guide cannulae were kept clean and covered using a screw-on dummy cannula (Plastics One). Mice were given 7 days to recover from surgery prior to ICV injections. All studies were carried out in accordance with United States National Institutes of Health guidelines and approved by the University of Illinois Institutional Animal Care and Use Committee.

Treatments were designed in a 2 × 2 factorial arrangement and administered at the onset of the dark cycle. ICV injections were administered using a 10 μl gas-tight syringe attached to internal injector cannulae (Plastics One) that extended 1 mm beyond the tip of the guide cannula, thus penetrating the lateral ventricle. All mice received treatments in 1 μl injection volume over a 1-min time period followed by an additional 1-min delay to allow diffusion before removing the injector cannula. Mice were injected ICV with either saline (control) or 300 ng/μl of zebularine (Sigma-Aldrich, St. Louis, MO, United States) and 30 min later with saline or LPS (10 ng/μl) from *Escherichia coli* O127:B8 (Sigma-Aldrich). This dose of LPS has been previously demonstrated to induce transient sickness behavior (Huang et al., 2008), and this dose of zebularine has been previously shown to induce behavioral changes in other mouse models (Anier et al., 2010).

### Burrowing Behavior

Decreased burrowing behavior is an indicator of sickness behavior (McLinden et al., 2012). Using a similar procedure described previously (Moon et al., 2015), burrows were constructed of polyvinyl chloride (PVC) pipe fitted at one end with a PVC pipe cap (closed end). The open end was raised 1.3 cm on twin steel legs. To acclimatize mice to the burrow, mice were singly housed in cages with the burrow present for 24 h prior to testing. Testing was initiated by adding 200 g of food pellets (Harlan Teklad 8640 chow, Madison, WI, United States) to the burrow immediately after the LPS/saline injections. Prior to replacing the burrow filled with food back into the cage, the burrow and food was weighed. Water was provided *ad libitum* but food was only available from the burrow. Mice were allowed to dig and/or eat the food out of the burrow for 48 h. Amount burrowed was calculated by subtracting the burrow and food weight before and 4, 8, 12, 24, and 48 h after burrowing. Body weight was also measured at baseline and 4, 8, 12, 24, and 48 h after the LPS/saline injections.

### Microglia Isolation

Microglia from mouse brains were isolated from separate groups of mice at 4 and 48 h after treatment. Mice were euthanized via CO_2_ asphyxiation, perfused with sterile ice-cold saline, and brain tissue (all but the hippocampus which was frozen on dry ice) was collected and used immediately for microglia isolation using a procedure adapted from Nikodemova and Watters (2012). Brains were enzymatically digested using the Neural Tissue Dissociation Kit (Miltenyi Biotec, Bergisch Gladbach, Germany) for 35 min at 37°C. Further processing was performed at 4°C. Tissue debris was removed by passing the cell suspension through a 40 μm cell strainer. After myelin removal using 30% Percoll Plus (GE Healthcare, Princeton, NJ, United States), cells in PBS supplemented with 0.5% BSA and 2 mM EDTA were incubated for 15 min with anti-Cd11b magnetic beads (Miltenyi Biotec). CD11b^+^ cells were extensively washed and separated in a magnetic field using MS columns (Miltenyi Biotec) before being directly placed in Trizol reagent (Invitrogen, Carlsbad, CA, United States).

### RNA Isolation and Real-Time RT-PCR

Total RNA from microglia and hippocampal tissue was isolated using the Tri Reagent protocol (Invitrogen). Synthesis of cDNA was carried out using a high-capacity RT kit (Applied Biosystems, Grand Island, NY, United States) according to the manufacturer’s instructions. Real-time RT-PCR was performed on an ABI PRISM 7900HT-sequence detection system (Perkin Elmer, Forest City, CA, United States). All genes were analyzed using commercially validated PrimeTime real-time RT-PCR Assays included in **Table 1** (Integrated DNA Technologies, Coralville, IA, United States), and were compared with the housekeeping control gene *Gapdh* using the 2^-ΔΔCt^ calculation method as previously described (Livak and Schmittgen, 2001). Data are expressed as fold change versus controls.

**Table 1:** Primers used in Real-time RT-PCR and Fluidigm experiments.

Gene	Assay ID
Arg1	Mm.PT.58.8651372
Casp1	Mm.PT.58.13005595
CD53	Mm.PT.58.30699738
CD68	Mm.PT.58.12034788.g
Cx3cr1	Mm.PT.58.17555544
Dnmt1	Mm.PT.58.30881142
Dnmt3a	Mm.PT.58.13545327
Dnmt3b	Mm.PT.58.31955137
Dnmt3l	Mm.PT.58.41749889
Gadd45b	Mm.PT.58.10699383.g
Gapdh	Mm.PT.39a.1
Gfap	Mm.PT.58.31297710
Gpr34	Mm.PT.58.46001700
Hdac1	Mm.PT.58.43356830.g
Hdac2	Mm.PT.58.12358619
Hdac3	Mm.PT.58.11480126
Hdac4	Mm.PT.58.17651425
Hdac5	Mm.PT.58.11472897
Hdac6	Mm.PT.58.16685964
*Il-1β*	Mm.PT.58.41616450
Il-1rn	Mm.PT.58.43781580
Il-10	Mm.PT.58.23604055
Il-4	Mm.PT.58.32703659
Il-6	Mm.PT.58.13354106
Mecp2	Mm.PT.58.13934895.g
Nlrp3	Mm.PT.58.13974318
P2ry12	Mm.PT.58.43542033
P2ry13	Mm.PT.58.42597879.g
Pycard	Mm.PT.56a.42872867
Siglech	Mm.PT.58.45915252
Socs1	Mm.PT.58.11527306.g
Socs3	Mm.PT.58.7804681
Stat3	Mm.PT.58.11877007
Tet1	Mm.PT.58.43326803
Tet2	Mm.PT.58.30089849
Tet3	Mm.PT.58.11954119
Tgfbr1	Mm.PT.58.10230349
Tlr2	Mm.PT.58.45820113
Tlr4	Mm.PT.58.41643680
Tlr7	Mm.PT.58.10526075
Tlr8	Mm.PT.58.16021150
Tmem119	Mm.PT.58.6766267
Trem2	Mm.PT.58.7992121
Tnf	Mm.PT.58.12575861

### Fluidigm

Total RNA from microglia collected at 4 h was isolated using the Tri Reagent protocol (Invitrogen) and synthesis of cDNA was carried out using a high-capacity RT kit (Applied Biosystems) as previously described for real-time RT-PCR. Fluidigm reactions were performed by the UIUC Functional Genomics Unit of the W. M. Keck Center using a 96 × 96 chip and included two technical replicates for each combination of sample and assay. Data was acquired using the Fluidigm Real-Time PCR Analysis software 3.0.2 (Fluidigm, San Francisco, CA, United States). Data from Fluidigm runs were manually checked for reaction quality before analysis, and *C*_t_ values for each gene target (see **Table 1**) were normalized to *C*_t_ values for the housekeeping gene *Gapdh*.

### DNA Isolation and Pyrosequencing

Total DNA from microglia and hippocampal tissue collected at both 4 and 48 h was isolated using the Tri Reagent protocol (Invitrogen). DNA methylation for 2 CpG sites within the proximal promoter region of *Il-1β* was assessed via bisulfite pyrosequencing on bisulfite modified DNA (Zymo Research, Irvine, CA, United States). Mouse *Il-1β* methylation assays (ID ADS3713-RS1) were purchased from EpigenDx (Hopkinton, MA, United States) and have been previously used to assess methylation status in microglia (Cho et al., 2015). PCRs were run in duplicate and contained 20 ng of bisulfite converted DNA as starting template. Product specificity was determined by gel electrophoresis. Each primer was also tested using bisulfite converted DNA from high and low methylation controls (EpigenDx). Qiagen’s PyroMark Q24 Advanced Pyrosequencer was used to detect DNA methylation levels following manufacturer’s protocols and default settings (Qiagen, Valencia, CA, United States), similar to a previous study (Bustamante et al., 2016).

### Statistical Analyses

All data were analyzed using GraphPad Prism 7 (La Jolla, CA, United States). Behavior and change in body weight data were analyzed via a two-way repeated-measures analysis of variance (ANOVA) for main effects of zebularine and LPS, and all two-way interactions. Gene expression and pyrosequencing data from primary microglia and hippocampus were subjected to a two-way ANOVA (no repeated measures) for main effects of zebularine and LPS, and all two-way interactions. Where analysis of variance revealed a significant interaction (*p* < 0.05 unless noted elsewhere), Tukey’s test was used for *post hoc* comparisons when appropriate. All data are expressed as means ± SEM.

## Results

### Body Weight Decreases With LPS in Both Saline and Zebularine Pre-treated Mice

**FIGURE 1:**
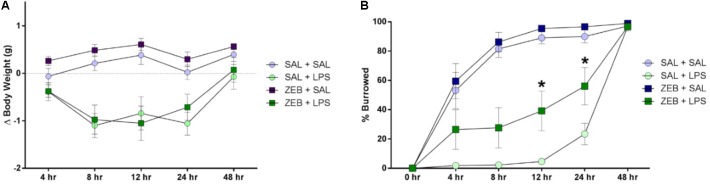
**(A)** Body weight was measured at baseline and 4, 8, 12, 24, and 48 h after SAL/LPS ICV injections in adult mice pre-treated with ICV SAL/ZEB. Data are presented as means ± SEM (*n* = 7–9). **(B)** Burrowing behavior was measured at 4, 8, 12, 24, and 48 h after SAL/LPS ICV injections in adult mice pre-treated with ICV SAL/ZEB. Data are presented as means ± SEM (*n* = 7–9). ^∗^*p* < 0.05.

Mice were either pre-treated ICV with saline or zebularine (ZEB), and then given an ICV injection of either saline (SAL) or LPS. For body weight, two way repeated measures ANOVA revealed main effects of both ZEB (*p* < 0.0001) and LPS (*p* < 0.0001), and an interaction (ZEB × LPS; *p* < 0.0001) (**Figure 1A**). *Post hoc* tests revealed that at 4 h, all treatment groups were not different (*p* > 0.05). At 8 and 12 h, the SAL + SAL group was different from SAL + LPS (*p* = 0.0002 and *p* = 0.0005) and ZEB + LPS (*p* = 0.0006 and *p* < 0.0001), and the ZEB + SAL group was also different from SAL + LPS (*p* < 0.0001 and *p* < 0.0001) and ZEB + LPS (*p* < 0.0001 and *p* < 0.0001), in that the SAL + LPS and ZEB + LPS groups lost more weight than the SAL + SAL and ZEB + SAL groups. At 24 h, the results were similar but the ZEB + LPS group was no longer different than the SAL + SAL group (*p* = 0.0651). By 48 h, body weight of the LPS-treated mice returned near baseline and all treatment groups were once again not different (*p* > 0.05).

### Zebularine Pre-treated Mice Recover Faster From Central LPS-Induced Sickness

For burrowing behavior, two way repeated measures ANOVA revealed main effects of both ZEB (*p* < 0.0001) and LPS (*p* < 0.0001), and an interaction (ZEB × LPS; *p* < 0.0001) (**Figure 1B**). *Post hoc* tests revealed that at 4 and 8 h, the SAL + SAL group was different from SAL + LPS (*p* < 0.0001 and *p* < 0.0001) and ZEB + LPS (*p* = 0.0474 and *p* < 0.0001), and the ZEB + SAL group was also different from SAL + LPS (*p* < 0.0001 and *p* < 0.0001) and ZEB + LPS (*p* = 0.006 and *p* < 0.0001), in that the SAL + LPS and ZEB + LPS groups burrowed less than the SAL + SAL and ZEB + SAL groups. At 12 and 24 h, the results were similar but the ZEB + LPS group was now different than the SAL + LPS group (*p* = 0.0071 and *p* = 0.0126), in that the ZEB + LPS was burrowing more. By 48 h, all of the mice burrowed out the majority of the food and treatment groups did not differ from one another (*p* > 0.05).

### Zebularine Alters LPS-Induced Expression of Inflammatory, Sensome, and Epigenetic Regulator Genes

**FIGURE 2:**
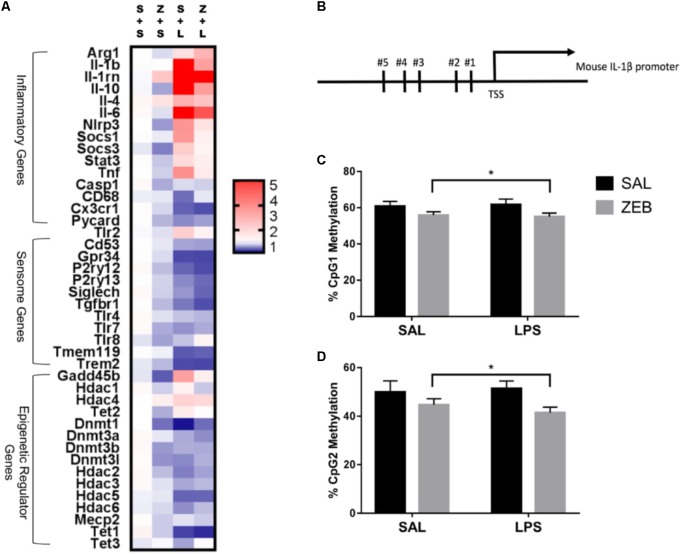
**(A)** Heat map visualization of relative expression of genes in microglia analyzed by Fluidigm. Microglia were collected at 4 h after SAL/LPS ICV injections in adult mice pre-treated with ICV SAL/ZEB. **(B)** Mouse *Il-1β* promoter. DNA methylation of the *Il-1β* promoter in microglia at **(C)** CpG1 and **(D)** CpG2. Data are presented as means ± SEM (*n* = 7–9). S + L, SAL + LPS; S + S, SAL + SAL; TSS, transcription start site; Z + L, ZEB + LPS; Z + S, ZEB + SAL. ^∗^*p* < 0.05.

Fluidigm gene expression analysis on microglia was performed to gain a more comprehensive assessment of microglial gene regulation in response to zebularine (**Figure 2A**). For inflammatory and regulators of inflammatory genes, there were a number of significant interactions in that ZEB decreased LPS-induced gene expression (*Il-1β*, *Il-1rn, Il-10, Nlrp3, Socs1, Tlr2*, and *Tnf*) (**Table 2A**). For sensome genes, there were no interactions of ZEB and LPS, but all were significantly or nearly significantly impacted by LPS, in that there were decreases compared to controls (**Table 2B**). Further, there were a few genes impacted by ZEB (*P2ry12, Siglech, Tgfbr1*) in that there were also decreases in gene expression. For epigenetic regulator genes, some *Dnmt*s decreased as expected (*Dnmt3a, Dnmt3b*), and *Hdacs* responded differently independently of HDAC family (i.e., *Hdac2, 3, 4*, and *5* had significant effects of LPS while *Hdac1* had a very significant effect of ZEB) (**Table 2C**). Of note, some epigenetic regulator genes were affected slightly different in the SAL + LPS group compared to what has been demonstrated with LPS IP injections in adults (*Dnmt1, Dnmt3b, Hdac1, Mecp2*) (Matt et al., 2016). For epigenetic regulators partially responsible for DNA demethylation, *Gadd45b* increased with LPS as expected, but was decreased by zebularine and there was an interaction that zebularine decreased LPS-induced expression. For gene expression of the Tet proteins, which are responsible for DNA hydroxymethylation and influencing DNA demethylation (Kohli and Zhang, 2013), *Tet1* and *Tet2* were effected by LPS and zebularine but LPS decreased *Tet1* and increased *Tet2*, while there was no effect of either treatment on *Tet3*.

**Table 2:** Expression of **(A)** inflammatory, **(B)** sensome, and **(C)** epigenetic regulator genes in microglia collected at 4 h after SAL/LPS ICV injections in adult mice pre-treated with ICV SAL/ZEB.

					*p-Values*		
Gene	SAL + SAL	ZEB + SAL	SAL + LPS	ZEB + LPS	ZEB	LPS	ZEB × LPS
**(A) Inflammatory and regulators of inflammatory genes**					
Arg1	1.05 0.32	0.88 0.22	1.80 0.71	2.96 0.85	0.467	0.046*	0.332
Casp1	1.16 0.08	0.72 0.05	0.88 0.06	0.84 0.07	0.001*	0.236	0.006*
Cd68	0.94 0.08	0.92 0.13	0.52 0.05	0.92 0.13	0.719	<0.0001*	0.567
Cx3cr1	1.08 0.05	0.86 0.07	0.48 0.08	0.42 0.08	0.056	<0.0001*	0.305
*Il-1β*	1.08 0.24	1.05 0.14	9.31 2.18	3.46 0.88	0.012*	<0.0001*	0.013*
Il-1rn	0.98 0.41	2.46 0.68	39.00 8.37	8.86 0.68	<0.0001*	<0.0001*	<0.0001*
Il-10	0.97 0.17	0.70 0.06	7.92 1.66	3.53 0.96	0.021*	<0.0001*	0.040*
Il-4	1.23 0.47	1.72 0.32	3.04 0.94	2.57 0.52	0.994	0.072	0.504
Il-6	1.14 0.15	0.89 0.09	10.31 1.71	5.38 1.20	0.034*	<0.0001*	0.055
Nlrp3	1.00 0.18	0.66 0.06	3.74 0.97	1.75 0.34	0.022*	0.0004*	0.098
Pycard	0.99 0.06	0.75 0.02	0.61 0.07	0.68 0.03	0.097	<0.0001*	0.004*
Socs1	0.99 0.13	0.93 0.08	3.66 0.46	1.37 0.23	<0.0001*	<0.0001*	<0.0001*
Socs3	0.92 0.13	0.58 0.06	2.33 0.30	1.27 0.27	0.003*	<0.0001*	0.11
Stat3	1.10 0.09	0.81 0.06	1.91 0.22	1.43 0.18	0.016*	<0.0001*	0.534
Tlr2	0.98 0.07	0.89 0.07	2.25 0.29	1.32 0.16	0.004*	<0.0001*	0.016*
Tlr4	1.12 0.07	0.89 0.07	0.80 0.06	0.76 0.04	0.035*	0.0005*	0.136
Tlr7	1.11 0.09	0.72 0.03	0.64 0.04	0.72 0.05	0.011*	0.0002*	0.0003*
Tlr8	0.90 0.19	0.69 0.12	0.80 0.12	1.28 0.17	0.414	0.133	0.038*
Tnf	1.01 0.17	0.84 0.11	3.86 0.58	1.54 0.28	0.0004*	<0.0001*	0.002*
**(B) Sensome genes**					
Cd53	1.01 0.18	0.92 0.19	0.70 0.06	0.68 0.16	0.738	0.081	0.8135
Gpr34	1.04 0.04	0.88 0.05	0.40 0.09	0.39 0.14	0.386	<0.0001*	0.447
P2ry12	1.14 0.09	0.78 0.08	0.49 0.09	0.41 0.12	0.035*	<0.0001*	0.17
P2ry13	1.03 0.07	0.85 0.07	0.61 0.09	0.51 0.09	0.0787	<0.0001*	0.657
Siglech	1.18 0.07	0.84 0.08	0.65 0.08	0.49 0.08	0.003*	<0.0001*	0.278
Tgfbr1	1.03 0.08	0.77 0.05	0.55 0.09	0.42 0.08	0.016*	<0.0001*	0.408
Tmem119	1.07 0.17	0.95 0.13	0.45 0.10	0.48 0.11	0.726	0.0003*	0.601
Trem2	0.92 0.13	0.77 0.20	0.40 0.07	0.39 0.10	0.541	0.001*	0.586
**(C) Epigenetic regulator genes**					
Dnmt1	1.00 0.18	0.53 0.14	0.24 0.09	0.52 0.14	0.524	0.012*	0.013*
Dnmt3a	1.17 0.13	0.92 0.04	0.72 0.13	0.62 0.07	0.041*	<0.0001*	0.369
Dnmt3b	1.17 0.10	0.66 0.06	0.73 0.20	0.72 0.09	0.044*	0.142	0.049*
Dnmt3l	1.10 0.33	0.64 0.11	0.61 0.16	0.74 0.13	0.397	0.324	0.137
Gadd45b	0.91 0.11	0.46 0.10	3.46 0.79	1.44 0.21	0.009*	0.0002*	0.015*
Hdac1	1.17 0.11	0.84 0.06	1.36 0.18	0.82 0.04	0.0002*	0.42	0.317
Hdac2	1.16 0.15	0.78 0.10	0.58 0.07	0.69 0.11	0.253	0.006*	0.041*
Hdac3	1.15 0.07	0.90 0.07	0.71 0.04	0.77 0.06	0.151	<0.0001*	0.015*
Hdac4	1.01 0.26	1.41 0.26	2.14 0.44	2.00 0.28	0.685	0.009*	0.39
Hdac5	0.95 0.16	0.92 0.18	0.49 0.09	0.49 0.11	0.921	0.003*	0.909
Hdac6	0.94 0.11	0.87 0.14	0.63 0.13	0.78 0.10	0.737	0.11	0.365
Mecp2	1.10 0.07	0.83 0.04	0.90 0.05	0.83 0.04	0.002*	0.071	0.054
Tet1	1.29 0.12	0.83 0.07	0.38 0.08	0.31 0.11	0.052	<0.0001*	0.2
Tet2	1.00 0.10	0.73 0.05	1.52 0.21	1.06 0.12	0.007*	0.002*	0.484
Tet3	0.92 0.20	0.98 0.23	0.7 0.21	1.07 0.33	0.496	0.836	0.615

*Data are presented as means ± SEM (*n* = 5–7) and *p*-values for main effects of ZEB and LPS as well as ZEB × LPS interactions are also included. ^∗^Indicates significance *p* < 0.05*.

### ICV Zebularine Decreases Methylation at CpG 1 and 2 of the *Il-1β* Promoter in Microglia at 4 h

DNA methylation for two CpG sites within the proximal promoter region of *Il-1β* was assessed via bisulfite pyrosequencing (**Figure 2B**). These two sites were chosen because they have been shown to be dynamically regulated in a previous study (Cho et al., 2015). For CpG 1, there was a main effect of ZEB (*p* = 0.0206), in that ZEB decreased DNA methylation (**Figure 2C**). For CpG 2, there was also a main effect of ZEB (*p* = 0.0228), in that zebularine decreased methylation (**Figure 2D**). There were no effects of LPS for either CpG site.

### Zebularine Altered LPS-Induced Expression of Inflammatory Genes and *Il-1β* Promoter Methylation in the Hippocampus at 4 h

**FIGURE 3:**
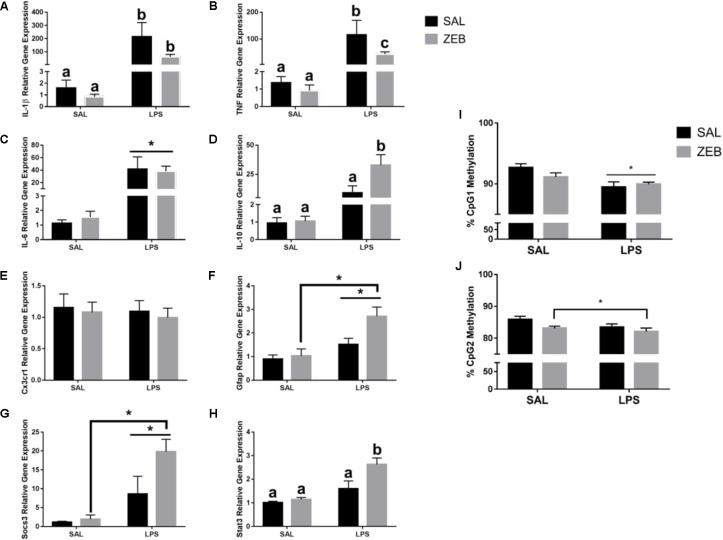
Hippocampal gene expression and DNA methylation at 4 h after SAL/LPS ICV injections in adult mice pre-treated with ICV SAL/ZEB. Data are presented as means ± SEM (*n* = 7–9) for **(A)** *Il-1β*, **(B)** *Tnf*, **(C)** *Il-6*, **(D)** *Il-10*, **(E)** *Cx3cr1*, **(F)** *Gfap*, **(G)** *Socs3*, and **(H)** *Stat3* gene expression. Data are presented as means ± SEM (*n* = 7–9) for DNA methylation of the *Il-1β* promoter in hippocampus at **(I)** CpG1 and **(J)** CpG2. Treatment means with different letters are significantly different. ^∗^*p* < 0.05.

In the hippocampus, for *Il-1β*, there were main effects of LPS (*p* = 0.001), ZEB (*p* = 0.0445), and an interaction (*p* = 0.0464) in that ZEB decreased LPS-induced *Il-1β* expression (**Figure 3A**). The same changes were observed for *Tnf*, with main effects of LPS (*p* < 0.0001), ZEB (*p* = 0.0362), and an interaction (*p* = 0.0384) (**Figure 3B**). For *Il-6*, there was only a main effect of LPS (*p* = 0.0005) (**Figure 3C**). For *Il-10*, main effects of LPS (*p* = 0.0016), ZEB (*p* = 0.0437), and an interaction (*p* = 0.0457) in that ZEB increased LPS-induced *Il-10* expression (**Figure 3D**). For *Cx3cr1*, no significant effects were observed (**Figure 3E**). For *Gfap*, main effects of LPS (*p* = 0.0006), ZEB (*p* = 0.033), and a trending interaction (*p* = 0.0815) in that ZEB increased LPS-induced *Gfap* expression (**Figure 3F**). Increased expression with ZEB and LPS was also seen for *Socs3* [LPS (*p* < 0.0001), ZEB (*p* = 0.0318), and a trending interaction (*p* = 0.0591)] and for *Stat3* [LPS (*p* < 0.0001), ZEB (*p* = 0.0083), and an interaction (*p* = 0.0347)] (**Figures 3G,H**). With regards to DNA methylation, for *Il-1β* CpG 1, there was a main effect of LPS (*p* = 0.0011), in that LPS decreased DNA methylation (**Figure 3I**). For *Il-1β* CpG 2, there was a main effect of ZEB (*p* = 0.0355), in that zebularine decreased methylation, and trending effect of LPS (*p* = 0.0844), in that LPS decreased methylation (**Figure 3J**).

### No Effect of Zebularine on Inflammatory Gene Expression at 48 h in Microglia and Hippocampus, but DNA Methylation Changes Still Present

**FIGURE 4:**
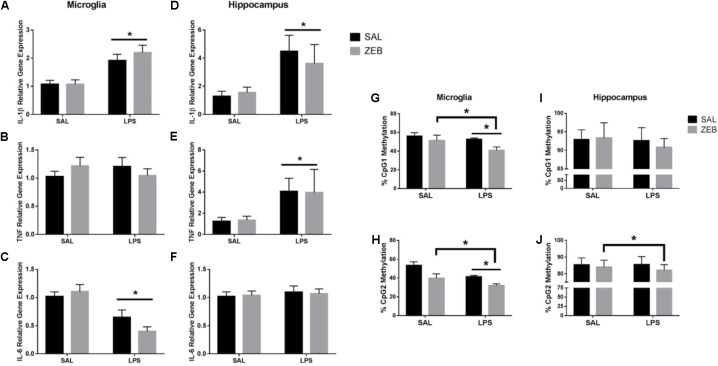
Gene expression and DNA methylation of microglia and hippocampus at 48 h after SAL/LPS ICV injections in adult mice pre-treated with ICV SAL/ZEB. Data are presented as means ± SEM (*n* = 7–9) for microglial **(A)** *Il-1β*, **(B)** *Tnf*, **(C)** *Il-6*, and hippocampal **(D)** *Il-1β*, **(E)** *Tnf*, and **(F)** *Il-6*. Data are presented as means ± SEM (*n* = 7–9) for DNA methylation of the *Il-1β* promoter in microglia at **(G)** CpG1 and **(H)** CpG2 and in hippocampus at **(I)** CpG1 and **(J)** CpG2. ^∗^*p* < 0.05.

Only main effects of LPS were observed at 48 h in both microglia and hippocampus (**Figures 4A–F**). For microglia, *Il-1β* and *Il-6* had main effects of LPS (*p* < 0.0001 and *p* < 0.001). For hippocampus, *Il-1β* and *Tnf* had main effects of LPS (*p* = 0.0052 and *p* = 0.0346). For *Il-1β* DNA methylation, in microglia, *Il-1β* CpG1 main effects of ZEB and LPS (*p* = 0.0186 and *p* = 0.0484) and *Il-1β* CpG2 main effects of ZEB and LPS (*p* = 0.0005 and *p* = 0.0021) in that ZEB and LPS decreased DNA methylation (**Figures 4G,H**). For hippocampus, *Il-1β* CpG1 trending main effect of LPS (*p* = 0.0844) and *Il-1β* CpG2 main effect of ZEB (*p* = 0.0341) in that ZEB and LPS decreased DNA methylation (**Figures 4I,J**).

## Discussion

The present study was designed to investigate a link between DNA methylation and neuroinflammation. We hypothesized that central administration of LPS and the DNMT inhibitor zebularine in adult mice would cause an exacerbated neuroinflammatory response, in that there would be exaggerated sickness behavior and a pro-inflammatory gene expression profile that would be associated with DNA demethylation of *Il-1β*. Surprisingly, instead of intensifying the LPS-induced sickness response and increasing inflammation in the brain, zebularine led to a quicker recovery of LPS-induced sickness behavior and decreased cytokine gene expression. Results of the Fluidigm analysis indicated that DNMT inhibition created an anti-inflammatory profile in microglia at 4 h, as many inflammatory genes were decreased by zebularine alone or in combination with LPS (*Il-1β, Tnf*, etc.). Although these results do not support the original hypothesis that pharmacological DNMT inhibition in the context of central immune activation causes an exaggerated cytokine response within the adult brain, similar to what has been seen *in vitro*, studies have demonstrated that DNMT inhibitors have an immunosuppresive effect in other cell types (Lee et al., 2015). Further, there is some indication that such anti-inflammatory effects of these drugs could translate to human disease, as patients with acute myeloid leukemia who were treated with a DNMT inhibitor after allogeneic stem cell transplantation were shown to have increased numbers of regulatory T cells, which was associated with a low incidence of graft-versus-host disease (Goodyear et al., 2012).

Novel findings using the Fluidigm analysis indicated differential expression of epigenetic regulators and sensome genes in microglia with central zebularine and LPS. Nearly all *Hdacs* analyzed were affected by LPS, a response that has been demonstrated elsewhere (Kannan et al., 2013). Further, *Hdac1*’s significant downregulation with zebularine supports the dependency of HDAC regulation on DNA methylation (Fuks, 2005). Of note, altered gene expression of Tet proteins, which has been explored very little in microglia, indicate a role of DNA hydroxymethylation in response to DNMT inhibition and LPS. To support this, it has recently been found that Tet2 is implicated in the regulation of cytokine expression during innate as well as T-cell-mediated immune responses, and that this is controlled by an HDAC-dependent mechanism (Zhang et al., 2015). For the majority of microglial sensome genes analyzed, they decreased with zebularine and/or LPS. As it has been demonstrated that these same genes are downregulated with aging and that it would be conceptually beneficial to the brain to downregulate the ability of microglia to get activated by dying or injured cells with aging (Hickman et al., 2013), reproducing this neuroprotective phenotype with DNMT inhibition during LPS-induced inflammation could indicate therapeutic potential in other inflammatory diseases.

Expression of inflammatory genes in the hippocampus at 4 h predominantly mirrored the anti-inflammatory changes in microglia. This included decreases in *Il-1β* and *Tnf* and increases in *Socs3* and *Stat3* in mice given both zebularine and LPS. There was an opposite effect of zebularine and LPS on *Il-10* expression, but this could be explained by the increases in *Gfap* expression. Increases in *Gfap* could indicate increased astrocyte activation and modulation of microglia within heterogeneous brain tissue (Villacampa et al., 2015; Lobo-Silva et al., 2016). Additional studies would be useful in identifying other cell populations such as astrocytes within the brain that are altered with DNMT inhibition during immune activation.

One particularly interesting finding was that although the effect of zebularine on gene expression disappeared at 48 h, DNA methylation changes were still present. The functions of DNA methylation in the brain have predominantly been investigated by exploring instances where changes in methylation within gene promoters correlate with changes in gene expression. Cases where basal levels of gene expression remain unaltered following a change in DNA methylation within the corresponding gene have been largely overlooked, which has led to a limited appreciation of the functional variations in DNA methylation across the genome (Baker-Andresen et al., 2013; Wagner et al., 2014). The relationship between DNA methylation and transcriptional activity is more complex than previously realized, and the relevance of an expanding repertoire of epigenomic modifications, particularly within the context of microglial development and activation across the lifespan, should to be explored. It is suggested that such epigenetic modifications may persist and alter the subsequent response to immune stimulation, thus providing a form of innate cell ‘memory’ that can contribute to a relatively non-specific resistance to re-infection — a phenomenon that has been termed ‘trained immunity’ (Netea et al., 2016). Experience-dependent variations in DNA methylation such as infection may prime the genome for response to later events by regulating transcription in response to incoming inputs, rather than by mediating enduring changes in gene expression.

## Conclusion

These data do support the hypothesis that epigenetic mechanisms like DNA methylation modulate neuroinflammatory responses. Perhaps the most exciting conclusion to be drawn from this work is that modulation of DNA methylation can influence not only the molecular features, but can also affect behavioral outcomes in animals. Pharmacological strategies aimed at specifically decreasing exacerbated microglial reactivity associated with infection might be important for improving recovery from sickness and reducing neurobehavioral deficits in populations like the elderly. More precise selectivity of epigenetic drugs is increasing, and with the recent generation of small-molecule inhibitors for multiple classes of histone modifiers, we will be better able to explore epigenetic proteins and uncover selective roles in the regulation of gene expression (Tough et al., 2014, 2016). The capacity of these compounds to selectively modify gene expression and to modulate pro-inflammatory responses indicates great promise for this approach in the treatment of human disease.

## Author Contributions

SM, ML, and RJ contributed conception of the study. SM and ML contributed design of the study. ML and AB contributed significant intellectual content in order to perform the experiments of the study. SM and JZ performed the actual experiments. SM performed the statistical analysis and wrote the first draft of the manuscript. ML and JZ wrote sections of the manuscript. RJ was responsible for the final approval of the submitted version. All authors contributed to manuscript revision, read, and approved the submitted version.

## Conflict of Interest Statement

The authors declare that the research was conducted in the absence of any commercial or financial relationships that could be construed as a potential conflict of interest.
